# Coronary inflammation and AI-Risk scores from cardiovascular computed tomography: impact on risk prediction and clinical management in a real-world setting

**DOI:** 10.1093/ehjimp/qyaf031

**Published:** 2025-04-17

**Authors:** John A Henry, Susannah M Black, Oliver G J Mitchell, Edward Richardson, Cameron Watson, Chris Hare, Pierre Le Page, Andrew R J Mitchell

**Affiliations:** Department of Cardiology, Jersey General Hospital, Gloucester Street, St. Helier JE1 3QS, Jersey; Oxford Centre for Clinical Magnetic Resonance Research, Division of Cardiovascular Medicine, Radcliffe Department of Medicine, University of Oxford, Oxford OX3 9DU, UK; Department of Cardiology, Jersey General Hospital, Gloucester Street, St. Helier JE1 3QS, Jersey; Department of Cardiology, Jersey General Hospital, Gloucester Street, St. Helier JE1 3QS, Jersey; Department of Cardiology, Jersey General Hospital, Gloucester Street, St. Helier JE1 3QS, Jersey; Department of Cardiology, Jersey General Hospital, Gloucester Street, St. Helier JE1 3QS, Jersey; Department of Radiology, Jersey General Hospital, Gloucester Street, St. Helier JE1 3QS, Jersey; Department of Cardiology, Jersey General Hospital, Gloucester Street, St. Helier JE1 3QS, Jersey; Department of Cardiology, Jersey General Hospital, Gloucester Street, St. Helier JE1 3QS, Jersey

**Keywords:** coronary artery disease, artificial intelligence, risk scores, perivascular inflammation

## Abstract

**Aims:**

Coronary computed tomography angiography (CCTA) is the primary investigation for stable chest pain. Despite approximately 80% of individuals undergoing CCTA not having obstructive coronary disease, this group contributes to two-thirds of major adverse cardiovascular events. Assessment of coronary inflammation using perivascular fat attenuation index (FAI) and AI-derived risk scores (AI-Risk) has demonstrated enhanced risk prediction beyond traditional clinical and CCTA parameters. We aimed to assess if FAI and AI-Risk alter risk prediction and clinical management in a real-world setting.

**Methods and results:**

Consecutive patients undergoing CCTA with FAI calculation and AI-Risk (CaRi-Heart®) at a single centre over a 3-year period were recruited. Conventional risk scores for non-fatal and fatal myocardial infarctions (QRISK3 and SCORE, respectively) were compared with AI-Risk. Clinical management decisions based on risk factors and CCTA results were recorded. FAI and AI-Risk scores were then provided and the resultant clinical management decision recorded. One hundred and sixty-four patients were included in the study (*n* = 164, male 78%, 56 years). Forty-eight per cent of the patients had no evidence of coronary artery disease (CAD) on CCTA, with 41% having non-obstructive CAD and 10% with potentially obstructive CAD. AI-Risk reclassified risk in 58% and 43% of patients compared with QRISK3 and SCORE, respectively. Clinical management was changed in 33% of patients following AI-Risk analysis.

**Conclusion:**

FAI and AI-Risk scores in a real-world setting changed risk prediction in around half of individuals and changed clinical management in around a third.

## Introduction

Coronary artery disease (CAD) remains one of the leading causes of death in Europe.^1^ Current standard of practice is to use risk calculators [e.g. QRISK3 and Systematic COronary Risk Evaluation (SCORE)] which focus on risk factors to identify those at risk of developing a myocardial infarction (MI). For those with suspected CAD, coronary computed tomography angiography (CCTA) is recommended as the first-line investigation to identify obstructive CAD.^2^ Recent data shows that as many as 80% of patients undergoing CCTA do not have obstructive CAD.^3^ Two-thirds of all MIs and just over 60% of cardiac deaths occur in these patients.^3^

It is now well recognized that inflammation plays a central role in the pathophysiology of atherosclerosis,^4^ and clinical trial evidence supports the use of anti-inflammatory treatments in secondary prevention.^5,6^ Imaging of perivascular coronary adipose tissue provides a window to their inflammatory state. Radiological changes in this adipose tissue can be quantified using the fat attenuation index (FAI), allowing estimation of coronary inflammation.^7,8^ Moreover, an artificial intelligence (AI) risk prediction model (AI-Risk) has been developed that includes FAI score alongside traditional risk factors (age, sex, smoking, diabetes, hypertension, and hypercholesterolaemia) and CCTA plaque burden to predict 8-year risk of a fatal cardiac event.^9^ Both FAI score and AI-Risk have been shown to predict major adverse cardiovascular events (MACE) and cardiac mortality independently of traditional risk factors and the presence or extent of CAD.^3^

Whether FAI score and AI-Risk, when applied independently in a real-world setting, changes risk prediction and clinical management is yet to be determined. This is what we sought to evaluate in this study.

## Methods

Consecutive patients undergoing CCTA with FAI and AI-Risk analysis at our centre between 28 April 2021 and 24 April 2024 were invited to take part in the study. FAI and AI-Risk assessment was automatically performed in all those with health insurance which covered the cost of the analysis. Patients referred for investigation of structural heart disease were excluded. The study was approved by the Jersey Research Ethics Committee (2024HCSREC04), and all patients included gave written informed consent.

Patient demographics, symptoms, risk factors, and medications were recorded from local healthcare records. This information was used to calculate a QRISK3 and SCORE risk prediction score to evaluate 10-year risk of non-fatal and fatal MI, respectively. CCTA scans were dual reported by a consultant cardiologist and radiologist each with >10 years CCTA-reporting experience, with a Coronary Artery Disease Reporting and Data System (CAD-RADS 2.0) score recorded.

CCTA scans were transferred to a cloud-based server for analysis with CaRi-Heart® v2.5 (Caristo Diagnostics, Oxford, UK) for analysis of FAI in each coronary artery, a percentile score for patient age and sex, and an AI-Risk score. The AI-Risk score incorporated the highest FAI score, plaque burden, and traditional risk factors as previously described.^3^ Patients were classified into manufacturer-recommended risk categories^3^ as follows:

- Low/medium-risk: AI-Risk < 5% and FAI score < 75th percentile in the LAD/RCA and <95th percentile in the LCx
- High-risk: AI-Risk 5% to <10% and/or FAI score in the LAD/RCA between 75th and 90th percentile and/or FAI score in the LCx > 95th percentile
- Very high-risk: AI-Risk ≥ 10% and/or FAI score at LAD/RCA > 90th percentile

Patient risk reclassification following FAI and AI-Risk relative to QRISK3 and SCORE was recorded. Clinical management decisions were made and recorded by the patient’s usual consultant cardiologist based on information from history, risk factors, and CCTA results. At least 48 h later, FAI and AI-Risk scores were supplied to the same clinician. Changes in patient management (statin initiation, escalation, or adjuvant treatment as previously suggested^3^) were then recorded by an independent clinician and compared with the previous decision pre-FAI and AI-Risk.

## Results

Of the 172 patients eligible to be included in the study, 164 provided written informed consent. Baseline demographics, risk factors, and medications are shown in *Table 1*. Of the whole cohort, 98 (60%) were conventionally classified as low risk (QRISK3 < 10%) with 79 (48%) having a 10-year risk of fatal MI (SCORE) < 2%. At baseline, 80 (49%) patients reported no chest pain and 41 (25%) reported breathlessness (*Table 1*).

**Table 1:** Baseline demographic and clinical characteristics

	Overall (*n* = 164)	No CAD CAD-RADS = 0 (*n* = 79, 48%)	Non-obstructive CAD CAD-RADS = 1, 2 (*n* = 68, 41%)	Potentially obstructive CAD CAD-RADS = 3,4,5 (*n* = 17, 10%)	Increase in risk classification^a^ (*n* = 56, 34%)	Change in management (*n* = 54, 33%)
Demographics						
Male, *n* (%)	128 (78%)	57 (72%)	59 (87%)	12 (71%)	46 (82%)	46 (85%)
Median age, years (IQR)	56 (50–62)	53 (49–59)	56 (50–62)	62 (56–69)	53 (49–85)	58 (52–63)
BMI, kg/m^2^	27.0 (3.9)	26.2 (3.9)	27.5 (3.7)	28.4 (4.4)	27.2 (3.6)	27.3 (3.4)
Cardiovascular risk factors, *n* (%)						
Smoker	9 (5%)	5 (6%)	3 (4%)	1 (6%)	4 (7%)	4 (7%)
DM	4 (2%)	2 (3%)	2 (3%)	0 (0%)	0 (0%)	4 (7%)
HTN	40 (24%)	14 (18%)	21 (31%)	5 (29%)	17 (30%)	22 (41%)
Hypercholesterolaemia	127 (77%)	59 (75%)	55 (81%)	13 (76%)	39 (70%)	38 (70%)
Total cholesterol, mmol/L (SD)	6.0 (1.2)	6.0 (1.1)	5.9 (1.2)	5.7 (1.1)	6.1 (1.2)	5.7 (1.3)
Total cholesterol/HDL ratio (SD)	4.3 (1.5)	4.4 (1.6)	4.4 (1.5)	3.8 (0.9)	4.4 (1.6)	4.2 (1.6)
QRISK3 score, *n* (%)						
Low/medium risk (<10%)	98 (60%)	55 (79%)	36 (53%)	7 (41%)	45 (80%)	25 (46%)
High risk (10–19%)	55 (34%)	22 (28%)	25 (37%)	8 (47%)	11 (20%)	24 (44%)
Very high risk (≥20%)	11 (7%)	2 (3%)	7 (10%)	2 (12%)	0 (0%)	5 (9%)
SCORE, *n* (%)						
<2%	79 (48%)	45 (57%)	29 (43%)	5 (29%)	36 (64%)	21 (39%)
2–5%	77 (47%)	31 (39%)	36 (53%)	10 (59%)	20 (36%)	31 (57%)
≥5%	8 (5%)	3 (4%)	3 (4%)	2 (12%)	0 (0%)	2 (4%)
Baseline medications, *n* (%)						
Antiplatelet	17 (10%)	6 (8%)	7 (10%)	4 (24%)	5 (9%)	10 (19%)
Statin	38 (23%)	15 (19%)	16 (24%)	7 (41%)	12 (21%)	16 (30%)
Symptoms, *n* (%)						
Breathless	41 (25%)	19 (24%)	14 (21%)	8 (47%)	17 (30%)	13 (24%)
Angina						
0	80 (49%)	36 (46%)	37 (54%)	7 (41%)	24 (43%)	27 (50%)
1	61 (37%)	31 (39%)	21 (31%)	9 (53%)	27 (48%)	20 (37%)
2	22 (13%)	12 (15%)	9 (13%)	1 (6%)	5 (9%)	7 (13%)
3	1 (1%)	0 (0%)	1 (1%)	0 (0%)	0 (0%)	0 (0%)

Angina score: 0 = no chest pain, 1 = non-cardiac sounding chest pain (one or zero key features), 2 = atypical chest pain (two features), and 3 = typical chest pain (three features). Key features: (1) precipitated by physical exertion; 2) constricting discomfort in the front of the chest, in the neck, shoulders, jaw, or arms; and (3) relieved by rest or glyceryl trinitrate (GTN) within about 5 min.

CAD, coronary artery disease (diagnosis based on the reported angiographic result of the index CCTA); CCTA, coronary computed tomography angiography; SCORE, Systematic COronary Risk Evaluation model and included age, sex, blood pressure, and smoking status; HDL, high-density lipoprotein; SD, standard deviation; IQR, interquartile range.

^a^Relative to QRISK3, which included age, sex, blood pressure, BMI, total cholesterol/HDL ratio, smoking, and diabetes status.

On CCTA assessment, 79 (48%) patients had no evidence of CAD (CAD-RADS 0), 68 (41%) had non-obstructive CAD (CAD-RADS 1 or 2), and 17 (10%) had potentially obstructive CAD (CAD RADS 3, 4, or 5). Average AI-Risk score was 2.5%. Eighty-five (52%) patients were categorized as low/medium risk, 49 (30%) as high risk, and 30 (18%) as very high risk based on FAI and AI-Risk.

Of the 79 patients with no evidence of CAD, 30 (38%) were categorized as high or very high risk following FAI and AI-Risk. Overall, 95 (58%) patients had their risk classification changed compared with QRISK3 (*Table 2*). Seventy (43%) had their risk category of fatal MI (<2%, 2–5%, ≥5%) changed compared with SCORE (*Table 3*). The demographic and clinical characteristics of those with increases in their risk classification are shown in *Table 1*.

**Table 2:** Reclassification table comparing AI-Risk classification to QRISK3

	AI-Risk classification				Reclassification upwards		Reclassification downwards	
QRISK3	Low/mid	High	Very high	Total				
Low/mid	53	27	18	98	45	46%		
High	29	15	11	55	11	20%	29	53%
Very high	3	7	1	11			10	91%
Total	85	49	30	164	56	34%	39	24%

**Table 3:** Reclassification table comparing AI-Risk of cardiac mortality to SCORE

	AI-Risk				Reclassification upwards		Reclassification downwards	
SCORE	<2%	2–5%	>5%	Total				
<2%	66	8	5	79	13	16%		
2–5%	32	28	17	77	17	22%	32	42%
>5%	2	6	0	8			8	100%
Total	100	42	22	164	30	18%	40	24%

**Figure 1:**
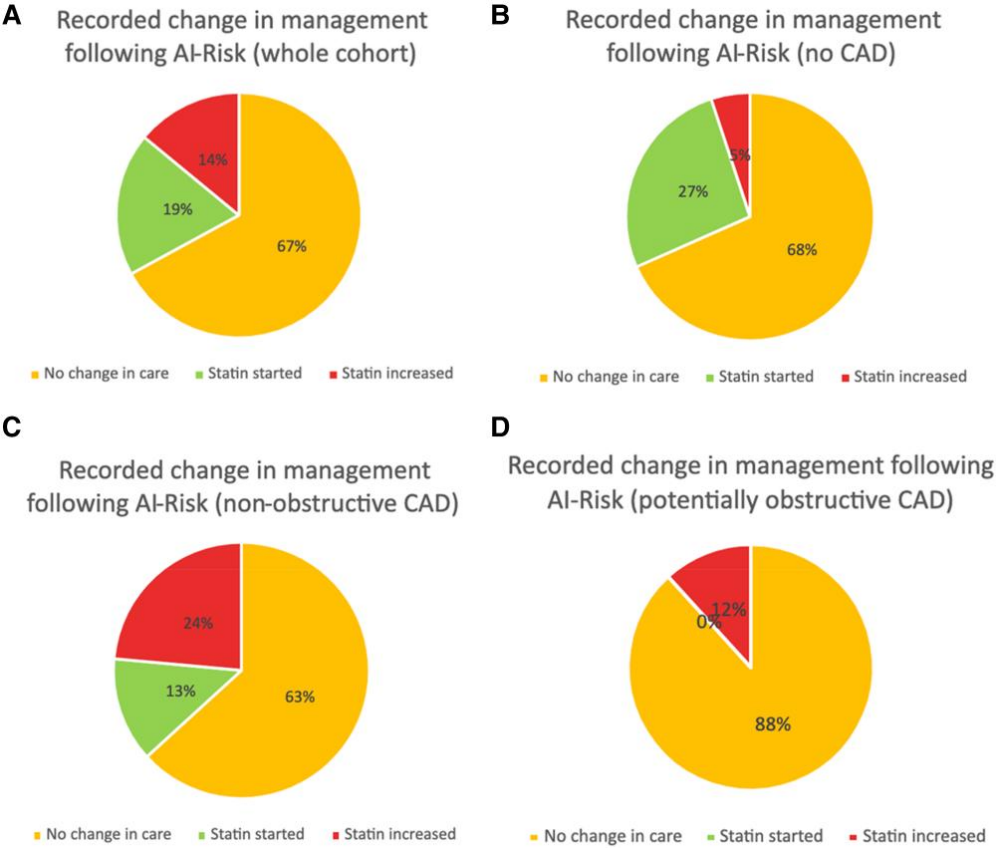
Recorded change in management following provision of AI-Risk results. (*A*) Whole cohort. (*B*) No evidence of CAD. (*C*) Non-obstructive CAD. (*D*) Potentially obstructive CAD.

In the cohort as a whole, 54 (33%) patients had their management changed following the provision of FAI and AI-Risk results (*Figure 1A*). In patients with no evidence of CAD, 25 (32%) had a change in their management (*Figure 1B*). For those with non-obstructive CAD, 25 (37%) had a change in their management (*Figure 1C*), while 2 (12%) patients with potentially obstructive CAD had a change in their management (*Figure 1D*). AI-Risk classification was significantly associated with change in treatment (*Table 4*). The demographic and clinical characteristics of those with changes in their management are shown in *Table 1*.

**Table 4:** Correlation between change in management and AI-Risk classification [*χ*^2^(4) = 65.57, *P* < 0.001]

	AI-Risk classification			
Treatment change	Low/mid	High	Very high	Total
No change	80	24	6	110
Statin initiation	3	14	14	31
Statin increase	2	11	10	23
Total	85	49	30	164

## Discussion

This study shows that FAI and AI-Risk, when applied in a real-world clinical setting, reclassified risk in over half of individuals compared with QRISK3, with a third of individuals having their risk category upgraded. AI-Risk also reclassified the risk of fatal cardiovascular events in 42% of individuals when compared with SCORE. These changes in risk classification translated to changes in clinical management, with one-third of individuals having their management altered following FAI and AI-Risk calculation.

These results are similar to those found by the developers of the technology. In a prospective evaluation survey of FAI and AI-Risk in 744 patients undergoing clinically indicated CCTA, they found management was changed in 45% of cases.^3^ Statin initiation or dose escalation occurred in 37% of patients, with adjuvant treatment strategies (e.g. colchicine, ramipril, aspirin, ezetimibe, and icosapent ethyl) accounting for the remaining 8%.^3^ It is unclear the extent of CAD in these individuals.

In this study, we show that calculation of FAI and AI-Risk has a significantly different effect on management across the spectrum of CAD severity. There were very few changes in management for those diagnosed with potentially obstructive CAD on CCTA. While FAI and AI-Risk have been shown to still predict MACE and cardiac mortality in those with obstructive CAD,^3^ this study questions whether this information leads to any meaningful changes in management.

The patients included in this study comprised a relatively low risk population, with 60% having a QRISK3 score < 10% and almost half having no demonstrable evidence of CAD on CCTA. Of these patients, we found that 38% had a high or very high risk classification following FAI and AI-Risk.

Importantly, it has previously been shown that patients with no or minimal CAD on CCTA but significantly elevated FAI score (≥75th percentile) have a 5-fold increase in MACE and almost 10-fold higher risk for cardiac mortality compared with those with the lowest FAI score (<25th percentile).^3^ Therefore, FAI and AI-Risk may represent an attractive method of identifying and potentially treating individuals with no or minimal CAD who are still at high risk due to coronary inflammation.

## Limitations

This study has a number of limitations. It is a relatively small, single-centre study with a population with low traditional risk scores and a largely reassuring symptom profile. It is possible that FAI and AI-Risk when applied to a higher risk population will result in more changes in risk prediction and clinical management. It should also be noted that no outcome data exist for FAI and AI-Risk directed changes in management, with current guidance coming from manufacturers of the technology. All treatment changes in this study were either statin initiation or dose escalation which are expected to indirectly target coronary inflammation through pleiotropic effects. Future studies are needed to investigate agents that directly target inflammation in those with high coronary inflammation. We were also unable to assess systemic markers of inflammation such as high-sensitivity C-reactive protein in this study. It is unclear how much value FAI and AI-Risk can add above widely available plasma inflammatory biomarkers.

## Conclusion

FAI and AI-Risk, when applied in a real-world clinical setting, lead to significant changes in risk prediction and clinical management, especially for those with no or non-obstructive CAD on CCTA. This technology may be useful in identifying and potentially treating patients who have traditionally been viewed as low risk of cardiovascular events but may go on to develop an event.

## Consent

Informed written consent was obtained from all participants.

## Data Availability

Data will be made available upon reasonable request.

## Lead author biography

Aaron Henry is an NIHR Academic Clinical Fellow in Cardiology at Oxford University Hospitals NHS Foundation Trust. He is also an Honorary Research Fellow at Jersey General Hospital. His research interests include multimodality cardiovascular imaging with a particular focus on obesity, cardiometabolic disease, and heart failure.
